# High consumption of ultra-processed foods is associated with increased risk of micronutrient inadequacy in children: The SENDO project

**DOI:** 10.1007/s00431-023-05026-9

**Published:** 2023-05-19

**Authors:** Lorena García-Blanco, Víctor de la O, Susana Santiago, Alba Pouso, Miguel Ángel Martínez-González, Nerea Martín-Calvo

**Affiliations:** 1 Olite Primary Care Health Center, Servicio Navarro de Salud-Osasunbidea, Pamplona, Spain; 2 IdiSNA, Instituto de Investigación Sanitaria de Navarra, Pamplona, Spain; 3 Precision Nutrition and Cardiometabolic Health. IMDEA Research Institute on Food & Health Sciences, Madrid, Spain; 4 School of Pharmacy and Nutrition, Department of Nutrition, Food Sciences and Physiology, University of Navarra, Pamplona, Spain; 5 Research Group on Community Nutrition and Oxidative Stress, University of the Balearic Islands, IDISBA & CIBERobn, 07122 Palma de Mallorca, Spain; 6 School of Medicine, Department of Preventive Medicine and Public Health, University of Navarra, C/ Irunlarrea 1, Pamplona, 31080 Spain; 7 Biomedical Research Centre Network on Obesity and Nutrition (CIBERobn), Physiopathology of Obesity and Nutrition, ISCIII, Madrid, Spain; 8 Department of Nutrition, Harvard T.H. Chan School of Public Health, Boston, USA

**Keywords:** Micronutrient, EAR, Ultraprocessed food, Healthy eating, Public health

## Abstract

Due to its rising prevalence, which parallels that of ultraprocessed food (UPF) consumption, inadequate micronutrient intake in childhood is a public health concern. This study aimed to evaluate the association between UPF consumption and inadequate intake of 20 micronutrients in a sample of children from the Mediterranean area. Cross-sectional information from participants in the “Seguimiento del Niño para un Desarrollo Óptimo” (SENDO) project 2015–2021 was used. Dietary information was gathered with a previously validated 147-item semi-quantitative food frequency questionnaire and the NOVA system was used to classify food items. Children were classified by tertiles of energy intake from UPF. Twenty micronutrients were evaluated, and inadequate intake was defined using the estimated average requirement as a cutoff. Crude and multivariable adjusted OR (95% CI) for the inadequacy of ≥ 3 micronutrients associated with UPF consumption were calculated fitting hierarchical models to take into account intra-cluster correlation between siblings. Analyses were adjusted for individual and family confounders. This study included 806 participants (51% boys) with a mean age of 5 years old (SD: 0.90) and an average energy intake from UPF of 37.64% (SD: 9.59). An inverse association between UPF consumption and the intake of 15 out of the 20 micronutrients evaluated was found (p < 0.01). After the adjustment for individual and family confounders, compared with children in the first tertile of UPF consumption, those in the third tertile showed higher odds of inadequate intake of ≥ 3 micronutrients (OR 2.57; 95%CI [1.51–4.40]).

*Conclusion*: High UPF consumption is associated with increased odds of inadequate intake of micronutrients in childhood.

**Table:** 

**What is Known:** *• Micronutrient deficiency is among the 20 most important risk factors for disease and affect around two billion people worldwide.* *• UPF are rich in total fat, carbohydrates and added sugar, but poor in vitamins and minerals.*	
**What is New:** *• Compared with children in the 1*^*st*^ *tertile of UPF consumption, those in the 3*^*rd*^ *tertile had 2.57 times higher odds (95% CI: 1.51-4.40) of inadequate intake of ≥ 3 micronutrients after adjusting for potential confounders.* *• The adjusted proportions of children with inadequate intake of ≥ 3 micronutrients were 23%, 27% and 35% in the 1*^*st*^, *2*^*nd*^*, and 3*^*rd*^ *tertiles of UPF consumption respectively.*

**Supplementary Information:**

The online version contains supplementary material available at 10.1007/s00431-023-05026-9.

## Introduction

Micronutrients are vitamins and minerals involved in several functions, including the production of enzymes, hormones, and other substances such as coenzymes, regulatory factors and antioxidants necessary for immunocompetence, normal growth and development. Although micronutrients are needed in very small amounts, they have a major impact on health [1]. An inadequate intake can not only cause visible and severe health conditions, but also less clinically notable reductions in energy level, mental clarity and overall capacity, leading to reduced educational outcomes (low level of concentration or attention, difficulties related to logical memory) and work productivity as well as increased risk of other diseases [1]. Indeed, micronutrient deficiency is among the 20 most important risk factors for diseases and affect around two billion people worldwide [2].

One way to reduce micronutrient deficiency is through an adequate intake of healthy food. However, ultra-processed foods (UPF) are penetrating dietary patterns across the globe [3]. Over the last 20 years, probably as a consequence of industrialization and globalization, the consumption of UPF has increased drastically worldwide, reaching the alarming proportion of 50%–60% of daily energy intake in several high-income countries [4–6]. Spain is not an exception since, in the last decade, in the whole population, the consumption of UPF increased from 11 to 32% [7, 8].

Cross-sectional and longitudinal studies in adult populations showed that UPF consumption is associated with higher body mass index [9–11] and waist circumference [11, 12], as well as higher risk of excess weight [10–13], hypertension [14], metabolic syndrome [15], dyslipidemias [16], asthma and wheezing [13], functional gastrointestinal disorders [17], overall cancer risk, and breast cancer risk [18]. These findings are at least partially explained by the nutritional quality of UPF-rich diets, that tend to be higher in total fat [19], carbohydrate [19, 20], Na [21], and added or free sugars [21, 22]. On the other hand, they tend to be lower in protein [19], fiber [19], vitamin C [19, 23, 24], vitamin A [19, 23], β-carotene [9], vitamin D [19, 23, 24], vitamin E [24], vitamin B_1_ [19, 23], vitamin B_2_ [23], vitamin B_6_ [19, 23], vitamin B_12_ [23], vitamin B_3_ [19, 23], folic acid [9], Zn [23, 24], K [19, 20, 23], P [23, 24], Mg [19, 23, 24], Ca [9, 23, 24], Fe [19, 23, 24], and fruits and vegetables [4, 9, 10, 24].

Although there are studies on the consumption of UPF and nutritional dietary profiles in the general population [25], to our knowledge, the association between UPF consumption and the risk of inadequate micronutrient intake in childhood has not yet been assessed. We hypothesized that children who have a high consumption of UPF may be at increased risk of having an insufficient intake of certain micronutrients. Therefore, we conducted this study to evaluate whether higher UPF consumption was associated with a higher number of micronutrients with suboptimal intake, as well as to calculate the marginal effect of UPF consumption on the risk of having an inadequate intake of ≥ 3 micronutrients in a sample of children from the Mediterranean area.

## Material and methods

### Study population

The “Seguimiento del Niño para un Desarrollo Óptimo” (SENDO) project (Follow-up of Children for Optimal Development) is a dynamic prospective pediatric cohort focused on studying the link between diet and lifestyle and the risk of childhood obesity. Pediatricians and our team’s researchers invite potential participants to join the study, through health care centers or schools. Through online self-administered questionnaires that parents complete, information is gathered at baseline and updated annually. Inclusion criteria are: 1) age between 4 and 5 years old, and 2) residence in Spain. The only exclusion criterion is the lack of an internet connected device to complete the questionnaires.

Out of the 989 children enrolled between 2015 and June 2021, 183 were excluded because they had not completed the baseline questionnaire at the start of the study. Therefore, the analyzed sample included 806 children with complete information in all the variables analyzed.

### Dietary information

Information on participants’ diets was gathered with a previously validated 147-item semi-quantitative food frequency questionnaire (FFQ) [26]. Parents reported how often their child had consumed each food item over the previous year by choosing one out of nine categories of response ranging from ‘never or almost never’ to ‘6 or more times per day’. A team of dietitians calculated the nutrient content of each food item multiplying the frequency of consumption by the edible portion and the nutrient composition of the pre-specified portion size. Information on nutrient composition was extracted from updated Spanish food composition tables [27].

All food items were classified by their degree of processing according to the NOVA classification system (Supplementary Table 1) [28, 29]. Four groups were defined: Group 1, unprocessed or minimally processed foods; Group 2, processed culinary ingredients; Group 3, processed foods; and Group 4, ultra-processed foods. The percentage of the total energy intake (TEI) that came from each NOVA group was calculated as:

$$(\text{energy contribution of each group/TEI})\times 100$$

We determined micronutrient intake adequacy for the following 20 micronutrients with known public health relevance: Zn; I; Se; Fe; Ca; K; P; Mg; Cr; Na; vitamin B1; vitamin B2; vitamin B3; vitamin B6; folic acid, vitamin B12; vitamin C; vitamin A; vitamin D, and vitamin E. The probability of intake adequacy was calculated by comparing the intakes of these nutrients with the estimated average requirements (EAR) if these were available or adequate intake (AI) levels, if not [30]. We used the traditional and probabilistic [31] approach, in which the probability of adequacy for the usual intake of a nutrient is calculated from a z-score, as:

$$\begin{aligned}\text{z-score}=&\;(\text{derived nutrient intake} - \text{EAR})/\text{standard deviation (SD) of the EAR}\end{aligned}$$

### Assessment of covariates

The questionnaire collected information on sociodemographic and lifestyle variables, family and personal medical history, and dietary habits.

Body mass index (BMI) was calculated using reported data as weight (kg)/height-squared (m^2^). Nutritional status was defined based on age and sex-specific cutoff points from the International Obesity Task Force [32]. Participants’ parents reported the time their child spent watching television, using the computer, or playing video games on average during the previous year. Information for weekdays and weekends was collected separately. Using that information, screen time was calculated as the mean hours/day a child spent watching television, using a computer, or playing video games. Information on physical activity was collected using a questionnaire validated for the Spanish population that included 14 activities and 10 response options ranging from never to 11 or more hours/week [33]. Four of these activities were rated as moderate (≤ 5 METs/hour), while 10 were rated as vigorous (> 5 METs/hour) [34] and the annual mean hours/day that each participant spent in moderate-to-vigorous activities was calculated. Questions about the recommended intake frequency of 18 distinct food groups and 9 response categories ranging from "Never" to "6 or more times per day" were used to assess parental knowledge of nutritional recommendations for children. Each question scored + 1 point if the answer met dietary recommendations and 0 points if it did not. Parents of participants were classified as having high (> 70%), medium (40–70%), or poor (40%) dietary awareness based on their final score. Through 8 yes/no questions, parental attitudes toward their child's eating habits were assessed. Positive responses (i.e., positive attitudes) scored + 1 point, whereas negative responses (i.e., negative attitudes) scored 0 points. Parents of participants were classified as having unhealthy (0–3 points), medium (4–6 points) or healthy attitudes (7–8 points) towards their child’s dietary habits.

### Statistical analysis

Participants were stratified in tertiles according to the dietary energy contribution of UPFs in their diet. We used numbers (percentages) for categorical variables and means (SD) for quantitative variables for descriptive purposes. By allocating the median of UPF consumption to each tertile and assuming this variable as continuous, linear trend tests across tertiles were calculated.

We calculated the association between UPF consumption (main independent variable) and 1) the mean number of micronutrients with inadequate intake (dependent variable in this model), and 2) the odds of inadequate intake of ≥ 3 micronutrients (dependent variable in this model). In subsequent analyses we evaluated the marginal effect, that is, the adjusted proportion of children with inadequate intake of ≥ 3 micronutrients in each tertile of UPF consumption. Crude and multivariable adjusted estimates were calculated. Multivariate analyses were progressively adjusted for the main known confounders according to the existing literature: model 1) sex, age, nutritional status, total energy intake; model 2) variables in model 1 plus maternal age, maternal higher education, parental knowledge about child nutritional recommendations, parental healthy dietary attitudes (healthy or unhealthy); and 3) variable in model 2 plus moderate-vigorous physical activity and screen time. We fitted hierarchical models (generalized estimating equations), which allowed the intra-cluster correlation between siblings to be taken into account. All p values are two-tailed. Statistical significance was settled at the conventional cut-off point of p < 0.05. Analyses were carried out using Stata version 15.0 (Stata Corporation).

### Ethical standard

The SENDO project was approved by the Ethics Committee for Clinical Research of Navarra (Pyto. 2016/122). Informed consent was obtained from the parents of all participants at enrollment.

## Results

This cross-sectional study included 806 participants (51% boys) with a mean age of 5 years old (sd 0.9) and an average UPF consumption of 38% (SD 9.5) of TEI. Medians (interquartile ranges) of UPF consumption by tertiles were 29% (24%-31%), 38% (36%-40%) and 46% (44%-52%) of TEI in T1, T2 and T3 respectively. Table 1 shows participants’ and families’ main characteristics according to tertiles of UPF consumption. Children who reported higher UPF consumption had slightly older mothers (p = 0.02) and came from larger families (p < 0.001). On the other hand, both parental attitudes towards their child’s dietary habits and parental knowledge on nutritional recommendations for children were inversely associated with UPF consumption (p < 0.001). Regarding children’s characteristics, those who reported higher UPF consumption were slightly older (p < 0.001), more physically active (p < 0.001), and spent more time on screens (p < 0.001). In contrast, they were less likely to have been breastfed (p < 0.001). A direct and marginally significant association was observed between UPF consumption and z-score of the BMI (p = 0.07). Nevertheless, mean z-scores of BMI were close to zero in all the tertiles of UPF consumption and absolute differences were small.

**Table 1:** Characteristics of participants and their families in the SENDO project by tertiles of UPF consumption. Numbers are mean (SD) or N (%)

	All the sample	T1	T2	T3	p for trend
N	806	269	269	268	
Median (interquartile range) of the percentage of total energy intake from UPF, %	38% (31%-44%)	27% (24%-31%)	38% (36%-40%)	48% (44%-52%)	

Family’s characteristics					
Maternal age (y)	40.0 (4.0)	39.6 (3.9)	40.0 (4.0)	40.4 (3.9)	0.02
Maternal age, N (%)					0.02
< 35 years	83 (10)	36 (13)	25 (9.2)	22 (8.2)	
35–40 years	323 (40)	107 (40)	116 (43)	100 (37)	
40–45 years	317 (39)	103 (38)	100 (37)	114 (43)	
> 45 years	83 (10)	23 (8.5)	28 (10)	32 (12)	
Maternal higher education, N (%)	649 (81)	217 (81)	228 (85)	204 (76)	0.2
Number of children, N (%)					< 0.001
1 child	87 (11)	39 (14)	21 (7.8)	27 (10)	
2 children	412 (51)	160 (60)	133 (49)	119 (44)	
3 children	179 (22)	54 (20)	60 (22)	65 (24)	
4 or more	128 (16)	16 (5.9)	55 (20)	57 (21)	
Family history of obesity, N (%)	157 (19)	56 (21)	50 (19)	51 (19)	0.59
Parental attitudes towards child’s dietary habits, N (%)					< 0.001
Unhealthy (0–3 p)	46 (5.7)	7 (2.6)	17 (6.3)	22 (8.2)	
Medium (4–5 p)	265 (33)	64 (24)	91 (34)	110 (41)	
Healthy (6–8 p)	495 (61)	198 (74)	161 (60)	136 (51)	
Parental knowledge about child’s nutritional recommendations, N (%)					< 0.001
Low (< 40%)	183 (23)	31 (12)	65 (24)	87 (32)	
Medium (40–70%)	518 (64)	183 (68)	168 (62)	167 (62)	
High (> 70%)	105 (13)	55 (20)	36 (13)	14 (5.2)	

Children’s characteristics					
Sex (female), N (%)	397 (49)	147 (55)	123 (46)	127 (47)	0.08
Age (y)	5.03 (0.9)	4.83 (0.7)	5.08 (0.9)	5.16 (0.9)	< 0.001
Race (white), N (%)	779 (97)	259 (96)	263 (98)	257 (96)	0.83
Gestational age, N (%)					0.77
< 38 weeks	117 (15)	37 (14)	41 (15)	39 (15)	
38–40 weeks	322 (40)	110 (41)	111 (42)	101 (38)	
≥ 40 weeks	361 (45)	120 (45)	114 (43)	127 (48)	
Birthweight (g)	3233 (552.2)	3215 (508.2)	3243 (519.2)	3242 (623.3)	0.56
Birthweight, N (%)					0.23
< 2,500 g	77 (9.6)	24 (8.9)	23 (8.6)	30 (11)	
2,500 to < 3,000 g	171 (21)	67 (25)	52 (20)	52 (19)	
3,000 to < 3,500 g	316 (39)	106 (40)	116 (44)	94 (35)	
3,500 to < 4,000 g	194 (24)	56 (21)	65 (24)	73 (27)	
≥ 4,000 g	42 (5.3)	14 (5.2)	10 (3.7)	18 (6.7)	
Breastfeeding duration, N (%)					< 0.001
No breastfeeding	125 (16)	18 (6.7)	49 (18)	58 (22)	
< 6 months	252 (31)	77 (29)	85 (32)	90 (34)	
6–12 months	209 (26)	68 (25)	71 (26)	70 (26)	
≥ 12 months	220 (27)	106 (39)	64 (24)	50 (19)	
Child’s position among siblings, N (%)					0.97
The oldest/ singletons	299 (37)	106 (39)	99 (37)	94 (35)	
2^nd^/3 or 2^nd^-3^rd^/4	138 (17)	27 (10)	61 (23)	50 (19)	
The youngest or > the 4^th^	369 (46)	136 (51)	109 (41)	124 (46)	
Z-score of the BMI	0.07 (1.2)	-0.04 (1.2)	0.12 (1.1)	0.13 (1.1)	0.07
Nutritional Status, N (%)					0.17
Low weight	113 (14)	46 (17)	36 (13)	31 (12)	
Normal weight	592 (73)	191 (71)	197 (73)	204 (76)	
Overweight/obesity	101 (13)	32 (12)	36 (13)	33 (12)	
Total energy intake (kcal)	2113 (538)	2043 (549)	2092 (486)	2205 (564)	< 0.001
Moderate-vigorous physical activity (h/day)	2.1 (2.7)	1.4 (1.3)	2.2 (2.7)	2.5 (3.4)	< 0.001
Screen time (h/day)	1.1 (0.9)	0.9 (1.0)	0.9 (0.6)	1.2 (0.8)	< 0.001

Table 2 shows energy-adjusted average intake of the 20 micronutrients evaluated by tertiles of UPF consumption. We found a significant inverse association between UPF consumption and the intake of 15 out of the 20 micronutrients evaluated (p < 0.01): vitamin A, vitamin C, vitamin D, vitamin E, vitamin B1, vitamin B3, vitamin B6, folic acid, vitamin B12, iron, phosphorus, magnesium, selenium, chrome, and potassium.

**Table 2:** Energy-adjusted micronutrient intake by tertiles of total UPF consumption

	T1	T2	T3	p for trend
N	269	269	268	
Micronutrients				
Vitamin A ($\mu$g/d)	1280 (31)	1132 (31)	1005 (31)	< 0.001
Vitamin C (mg/d)	180 (4.2)	145 (4.2)	119 (4.2)	< 0.001
Vitamin D ($\mu$g/d)	3.87 (0.12)	3.43 (0.12)	2.87 (0.12)	< 0.001
Vitamin E (mg/d)	10.1 (0.2)	8.73 (0.20)	7.82 (0.20)	< 0.001
Vitamin B_1_ (mg/d)	1.54 (0.01)	1.51 (0.01)	1.45 (0.01)	< 0.001
Vitamin B_2_ (mg/d)	2.14 (0.03)	2.13 (0.03)	2.18 (0.03)	0.37
Vitamin B_3_ (mg/d)	39.9 (0.5)	37.9 (0.5)	34.8 (0.5)	< 0.001
Vitamin B_6_ (mg/d)	2.59 (0.03)	2.39 (0.03)	2.21 (0.03)	< 0.001
Folic Acid ($\mu$g/d)	352 (5.2)	315 (5.2)	285 (5.2)	< 0.001
Vitamin B_12_ ($\mu$g/d)	5.09 (0.09)	4.96 (0.09)	4.71 (0.09)	0.004
Ca (mg/d)	1231 (17)	1226 (17)	1212 (17)	0.44
I ($\mu$g/d)	115 (1.4)	114 (1.4)	113 (1.4)	0.35
Fe (mg/d)	15.3 (0.1)	14.5 (0.1)	13.6 (0.1)	< 0.001
P (mg/d)	1972 (54)	1845 (54)	1779 (54)	0.01
Mg (mg/d)	333 (2.9)	306 (2.9)	293 (3.0)	< 0.001
Se ($\mu$g/d)	78.1 (0.8)	75.1 (0.8)	70.4 (0.8)	< 0.001
Zn (mg/d)	10.3 (0.1)	10.1 (0.1)	9.9 (0.1)	0.06
Cr ($\mu$g/d)	74.6 (1.5)	73.4 (1.5)	68.3 (1.5)	0.006
K (mg/d)	3837 (42)	3518 (42)	3313 (42)	< 0.001
Na (mg/d)	3069 (57)	3143 (57)	3073 (57)	0.93

Numbers are mean (SD)

**Fig. 1:**
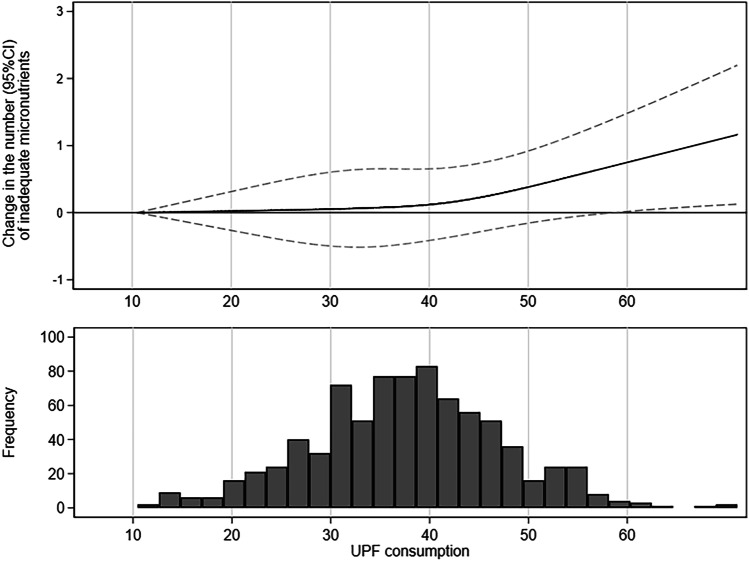
The spline above shows the difference (95% CI) with respect to the lowest consumption (10% of energy intake) in the average number of inadequate micronutrients associated with ultra-processed food consumption. The histogram below shows the frequency of participants by ultra-processed food consumption

After adjusting for potential confounders, the number of micronutrients with inadequate intake rose by 0.14 (95% CI 0.06–0.23) for each 10% increase in UPF consumption (data not shown). Figure 1 shows a direct association between UPF consumption and the average number of micronutrients with inadequate intake. The spline at the top of Fig. 1 shows the change in inadequate micronutrient intake (solid line) and 95% CI (dashed line) associated with one unit increase (% of TEI) in UPF consumption. The slope of the line becomes steeper for UPF consumption above 45% of TEI, but the association reaches statistical significance only for greater values of UPF consumption (approximately 60% of TEI). According to the histogram at the bottom of Fig. 1, the distribution of UPF consumption in this sample was almost normal, with a slight positive skewness. 70% of participants reported UPF consumption between 30 and 50% of TEI, a range in which one unit increase in the percentage of UPF consumption was not significantly associated with an increase in the number of inadequate intakes of micronutrients.

A significant linear trend was found in the odds of inadequate intake of ≥ 3 micronutrients across tertiles of UPF consumption (Table 3). Compared with children in the first tertile (T1), those in the third tertile (T3) of UPF consumption had 1.59 times higher odds (95% CI 1.09 – 2.33) of inadequate intake of ≥ 3 micronutrients in the crude model. Stronger estimates were observed through progressive adjustments. The most adjusted model showed that, after accounting for personal (sex, age, nutritional status, energy intake, physical activity and screen time) and family confounders (maternal age, maternal higher education, parental knowledge on nutritional recommendations in childhood and parental attitudes towards their child’s dietary habits), children in T3 of UPF consumption had 2.57 times higher odds (95% CI: 1.51–4.40) of inadequate intake of ≥ 3 micronutrients compared with their peers in T1.

**Table 3:** OR and 95% Confidence Interval (CI) of failing to meet 3 or more micronutrients recommendations associated with the consumption of ultra-processed foods (% of TEI)

	OR (95% CI)			P for trend
	T1	T2	T3	
N	269	269	268	
N of participants (%) failing to meet ≥ 3 micronutrients recommendations	64 (24)	74 (28)	89 (33)	
Crude	1.00 (Ref.)	1.26 (0.87–1.85)	1.59 (1.09–2.33)	0.017
Multivariable adjusted model 1	1.00 (Ref.)	1.47 (0.92–2.35)	2.99 (1.81–4.95)	< 0.001
Multivariable adjusted model 2	1.00 (Ref.)	1.30 (0.80–2.12)	2.52 (1.50–4.26)	0.001
Multivariable adjusted model 3	1.00 (Ref.)	1.41 (0.86–2.32)	2.57 (1.51–4.40)	0.001

Model 1 is adjusted for sex (male or female), age (continuous), nutritional status (underweight, normal weight, overweight/obese), total energy intake (tertiles)

Model 2 is additionally adjusted for maternal age (< 35y, 35–40y, > 40–45y, > 45y), maternal higher education (yes or no), parental knowledge about child’s nutritional recommendations (low, medium score or high), parental attitudes towards child’s dietary habits (unhealthy, average, healthy)

Model 3 is additionally adjusted for physical activity (tertiles) and screen time (tertiles)

**Fig. 2:**
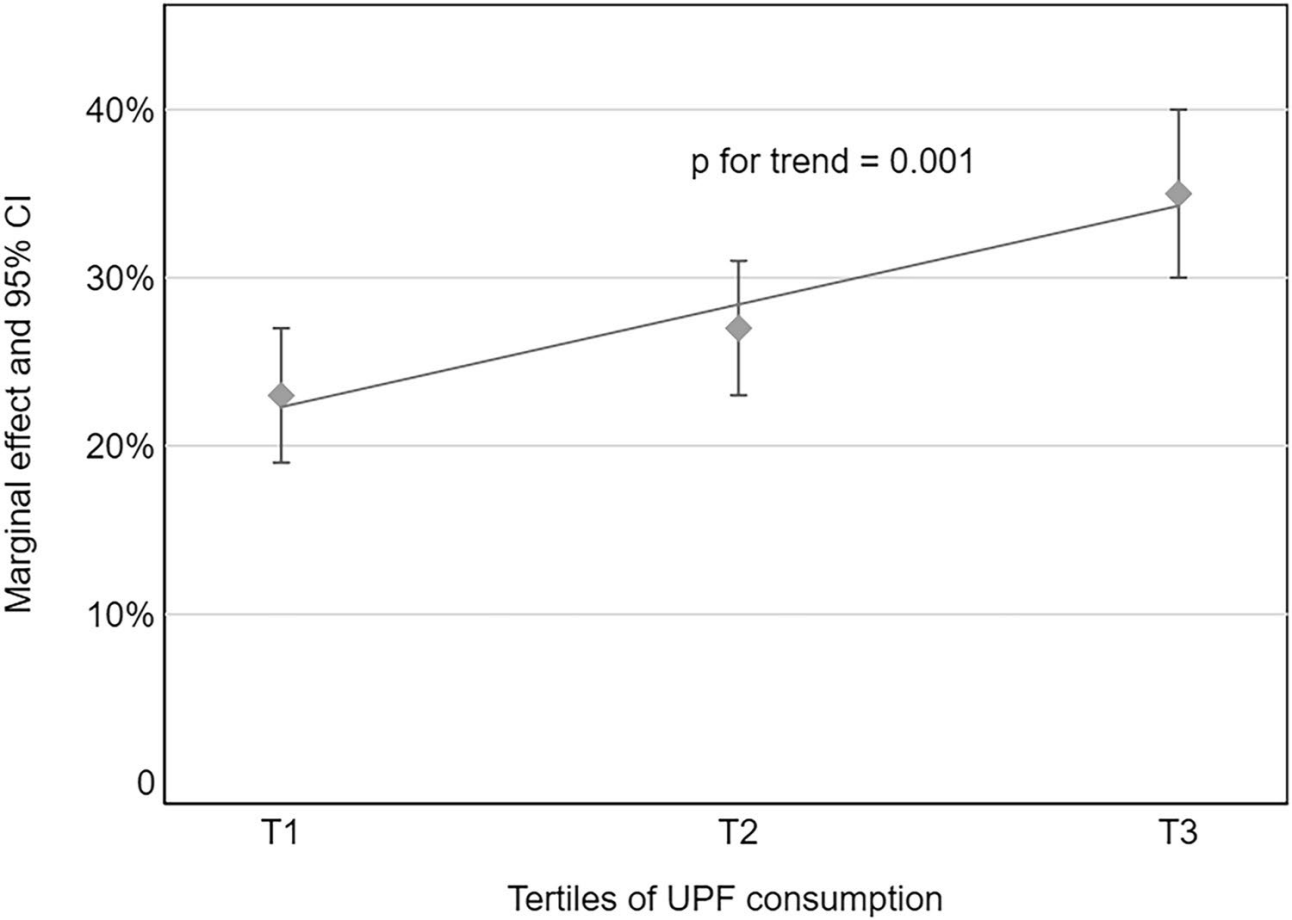
Adjusted proportions (95% CI) of children with 3 or more micronutrient inadequacy by tertiles of UPF consumption using the traditional method to define inadequacy

After accounting for all the potential confounders, the adjusted proportions of children with inadequate intake of ≥ 3 micronutrients were 23% (95% CI: 19%-27%), 27% (95% CI: 23%-31%) and 35% (95% CI: 30%-40%) in T1, T2 and T3 respectively. Figure 2 shows the point estimates (95% CI) and the fitting line with a slope of 0.58, which indicates that 1% increase in UPF consumption was associated with a 0.58 increase in the percentage of children with inadequate intake of 3 or more micronutrients (p for trend < 0.001).

To assess the strength of our findings, we re-ran the analyses after excluding children with extreme values of energy intake (above 99^th^ percentile and below 1^st^ percentile) and obtained similar estimates (data not shown). On the other hand, we observed a reduced proportion of children with inadequate intake of ≥ 3 micronutrients using the probabilistic approach. Moreover**,** sensitivity analysis using the probabilistic approach also showed a significant trend for inadequate intake of 2 or more micronutrients across tertiles of UPF consumption (Supplementary Fig. 1).

## Discussion

In this cross-sectional analysis of children in the SENDO project a direct linear association between UPF consumption and the mean number of micronutrients with inadequate intake was found, with significant increases for UPF consumption above 60% of TEI. Indeed, after accounting for personal and family potential confounders, children in the third tertile of UPF consumption showed higher odds (OR 2.57; 95% CI: 1.51- 4.40) of failing to comply with ≥ 3 micronutrient recommendations. Along with this, we found that the proportion of children with inadequate intake of ≥ 3 micronutrients significantly increased across tertiles of UPF consumption from 23% (95% CI: 19%-27%) in T1 to 35% (95% CI: 30%-40%) in T3. Previous evidence focused on single micronutrients or on small number of them [35]. To the best of our knowledge, the present study is the first to investigate the association between the consumption of UPF and the intake of 20 micronutrients with public health relevance in children from the Mediterranean area.

We consider these findings to be of great interest because they reinforce the need for public health strategies to eliminate UPF from children's diets [36, 37]. Although we found significant increases in the number of inadequate micronutrients for UPF consumption over 60%, that threshold must be interpreted with caution. We reported with good reason the marginal effect of UPF consumption, which is easier to interpret and to convey to the population [38]. Although that threshold is far from the mean UPF consumption observed in our sample, several high-income countries have reported mean UPF consumption above 50–60% of TEI [5]. The high proportion of children with inadequate intake of ≥ 3 micronutrients in all the tertiles of UPF consumption shows that there is no place for UPF in a healthy diet and that we should not concern ourselves with defining a healthy threshold. The Big Food companies invest heavily in studying the texture, taste, smell, shape, sound in the mouth and packaging of UPF in order to generate highly attractive, addictive and ultra-palatable products, to which it is very difficult not to succumb [39]. The marketing of UPF is also an issue of concern. Almost 80% of food advertisements are for UPF [40]. Besides, the advertising departments of these companies very often include psychologists and neurologists that know how to influence our conscious and unconscious decision making [41]. In addition, there is the issue of accessibility and affordability. According to the Ministry of Agriculture, Fisheries and Food [42], in Spain most food products (74%) are purchased in supermarkets or hypermarkets, where the supply of unprocessed or minimally processed products is lower than that of UPFs (20% compared to approximately 80%), which also tend to have more and better economic offers. There is an urgent need to regulate the production, manufacturing, advertising and distribution of this type of product while implementing initiatives that favor the consumption of fresh foods to facilitate the adoption of healthier diets by families [43].

Our team previously published that parents’ healthy attitudes towards their child’s dietary habits were associated with greater nutritional adequacy of the child’s diet [44] and that they are a strong predictor of UPF consumption [45]. The present study adds to existing evidence by suggesting that higher UPF consumption may be the link between unhealthy parental attitudes toward the child’s dietary habits and nutritional inadequacy. Although prospective studies are needed before causality can be inferred, this evidence suggests that the observed association is probably not just due to residual confounding.

Our data agree with previous studies regarding the mean UPF consumption and the prevalence of micronutrient inadequacy [46]. Nevertheless, we acknowledge that the use of self-reported data might have hampered our results and that our estimates may represent the upper limit of the true association. Further research is needed to elucidate the real magnitude of the risk of micronutrient deficiency associated with UPF consumption.

Our findings contrast with a previous study in Brazilian children that reported that 2 to 3-year-old children from low-income families could be at risk of excessive micronutrient intakes provided by UPF [47]. However, it must be noted that that study focused on fortified products for young children, which do not represent the whole group of products in NOVA 4 group.

We must acknowledge some limitations. First, due to the cross-sectional design, causality cannot be inferred. Second, FFQ used in the SENDO project did not collect information on food processing. To reduce possible misclassification, two researchers categorized food items into the NOVA groups and discrepancies were resolved by consensus. Third, although FFQ are the most practical and feasible tool to measure dietary intake in epidemiological studies [48], they tend to overestimate dietary intake and therefore they cannot be considered the gold standard to calculate micronutrient intake. An overestimation of dietary intake in this study would mean that the micronutrient inadequacy may still be higher than observed. Fourth, our results only show the probability of nutritional adequacy, since the best way to assess actual nutrient deficiency would be through the determination of biomarkers of nutrient intake. Fifth, most of the participants in the SENDO project have highly educated parents and live in a developed country. Although this factor may limit the generalizability of our findings, it reduces the confounding by socio-economic status [49]. Sixth, given the observational design of this study, the possibility of residual confounding by unmeasured variables cannot be completely ruled out. Lastly, the questionnaire on parental knowledge on nutritional recommendations for children and the one on their attitudes towards their child’s dietary habits have not been validated. However, previous studies showed that they are associated with diet quality in adult populations [50, 51].

Our study has several strengths, including the relevance of the issue and the fact that dietary intake of 20 micronutrients was assessed. Additionally, the sample size was large, and the questionnaire was extensive, which enabled adjustment for several personal and family confounders. Besides, analyses were fitted with hierarchical models that took into account intra-cluster correlation between siblings, a prevalent flaw in research on pediatric populations.

In conclusion, we found that high consumption of UPF is associated with increased odds of inadequate intake of ≥ 3 micronutrients in childhood. Although our sample reported an average UPF consumption of 38%, which is lower than the alarming 50–60% observed in several high-income countries, we still found a direct linear association between UPF consumption and micronutrient inadequate intake. Public policies and health education initiatives are needed to eliminate UPF from children’s diet**s** and foster the consumption of unprocessed and minimally processed products.

## Supplementary Information

Below is the link to the electronic supplementary material.

Supplementary file1 (TIF 1575 KB)

Supplementary file2 (DOCX 14 KB)

## Data Availability

The datasets generated and/or analyzed during the current study are available from the corresponding author on reasonable request.

## List of abbreviations in alphabetical order

BMI: Body mass index
Ca: Calcium
CI: Confidence interval
Cr: Chrome
EAR: Estimated Average Requirement
Fe: Iron
FFQ: Food-frequency questionnaire
I: Iodine
K: Potassium
Mg: Magnesium
Na: Sodium
OR: Odds Ratio
P: Potassium
RDA: Recommended Dietary Allowance
SD: Standard deviation
Se: Selenium
SENDO: Seguimiento del Niño para un Desarrollo Óptimo
T1-T2-T3: First, second and third tertiles
TEI: Total energy intake
UPF: Ultra processed foods
Zn: Zinc
