# Supplementary figures and images for: High consumption of ultra-processed foods is associated with increased risk of micronutrient inadequacy in children: The SENDO project
Source: Eur J Pediatr. 2023 May 19;182(8):3537–47. doi: 10.1007/s00431-023-05026-9 (PMC10460344; doi:10.1007/s00431-023-05026-9)

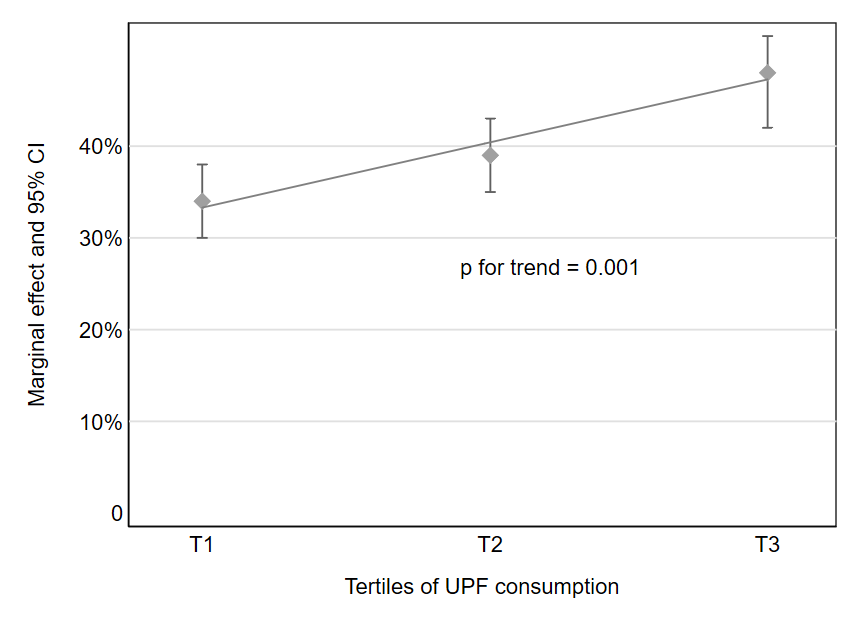

Supplement: Supplementary file 1 — Supplementary file1 (TIF 1575 KB) [file 431_2023_5026_MOESM1_ESM.tif]
