# Supplementary material for: High consumption of ultra-processed foods is associated with increased risk of micronutrient inadequacy in children: The SENDO project
Source: Eur J Pediatr. 2023 May 19;182(8):3537–47. doi: 10.1007/s00431-023-05026-9 (PMC10460344; doi:10.1007/s00431-023-05026-9)
Supplement: Supplementary file 2 — Supplementary file2 (DOCX 14 KB) [file 431_2023_5026_MOESM2_ESM.docx]

**Supplementary table 1: Classification on foods in the SENDO food frequency questionnaire according to the** **extent and purpose of processing by NOVA.**

| **Unprocessed or minimally processed foods** | **Processed culinary ingredents** | **Processed foods** | **Ultra-processed food and drink products** |
| --- | --- | --- | --- |
| **Apple, asparagus, eggplant, avocado, banana,** , beans, cabbage, carrot, chard, cherry, chicken, clam, curd, eggs, fig, fish, fruit juice, fruit smoothie, garbanzo beans, grapes, kiwi fruit, lamb, leek, lentils, lettuce, mango, meatball, melon, milk (skimmed or whole), nuts, octopus, onion, orange, pasta, peas, peach, pear, pepper, pineapple, plum, pork, potatoes, seafood, pumpkin, rabbit meat, rice, strawberry, string beans, tangerine, tomato, veal, viscera,watermelon | Sunflower oil, olive oil, sugar, butter, cream, salt. | Olives, compote of fruit, cured ham, canned fish, jam, baguette, wholemeal bread, white cheese, cured cheese,bacon | Bakery products, blood sausage, bonbon, breakfast cereals, cake, candies, carbonated beverages, cereal bar, chocolate bar, chocolate powder, cookies, crab sticks, cream cheese, cream chocolate, croquet*, cruller or ‘churro’*, custard*, dry soup, fish sticks, gelatine, ham, hamburger, ice cream, industrialised juices (sugar-sweetened juices), industrialised sliced cheese, industrialised sliced bread, ketchup, lasagne*, margarine, mayonnaise*, muffin*, nougat, nugget, pâté, petit suisse, pie*, pizza*, popcorn*, salami, sausage, pepperoni, snacks, soda, soft drinks, sweetened beverages, sweetened fermented milk, sweetened yogurt (skimmed or whole) |

*There are foods that could have different ratings depending on the way they are prepared: homemade or industrialised. In these cases, we chose to classify them as ultra-processed foods because most traditional foods have been replaced by industrial food products in supermarkets
